# EXAMINING COVID-19 VACCINE–RELATED AGEISM IN TWITTER DATA

**DOI:** 10.1093/geroni/igad104.0203

**Published:** 2023-12-21

**Authors:** Juanita-Dawne Bacsu, Megan O’Connell, Allison Cammer, Alison Chasteen, Sarah Fraser, Mehrnoosh Azizi, Karl Grewal, Raymond Spiteri

**Affiliations:** Thompson Rivers University, Kamloops, British Columbia, Canada; University of Saskatchewan, Saskatoon, Saskatchewan, Canada; University of Saskatchewan, Saskatoon, Saskatchewan, Canada; University of Toronto, Toronto, Ontario, Canada; University of Ottawa, Ottawa, Ontario, Canada; University of Saskatchewan, Saskatoon, Saskatchewan, Canada; University of Saskatchewan, Saskatoon, Saskatchewan, Canada; University of Saskatchewan, Saskatoon, Saskatchewan, Canada

## Abstract

During the pandemic, many high-income countries prioritized older adults for COVID-19 vaccination in an attempt to reduce mortality. This prioritization may have exacerbated ageism and intergenerational conflict, especially with the limited quantity of COVID-19 vaccines. This presentation examines vaccine-related ageism during COVID-19 on social media to inform future vaccination campaigns and policies. Using Twitter, we gathered 1,369 relevant tweets using the Twint application in Python from December 8, 2020 to December 31, 2021. Tweets were assessed using inductive thematic analysis and steps were taken to ensure rigor and trustworthiness. Based on our analysis, four main themes were identified including: i) Blame and aggression; ii) Misinformation and mocking content; iii) Ageist political insults; and iv) Challenging ageism. Our study identified issues of false information, hate speech, and ageist political insults that are contributing to intergenerational conflict. Although some tweets challenged this derogatory messaging and demonstrated intergenerational unity, our findings suggest ageism contributed to COVID-19 vaccine hesitancy among older adults. Accordingly, urgent action is required to challenge vaccine misinformation, counter aggressive ageist content, and support intergenerational unity during the pandemic.

